# A Pandemia de COVID-19 e a Doença Cardiovascular no Brasil: Aprendendo com os Dados

**DOI:** 10.36660/abc.20220371

**Published:** 2022-07-07

**Authors:** Fernando Cesena

**Affiliations:** 1 Cenocor Guarulhos SP Brasil Cenocor, Guarulhos, SP – Brasil

**Keywords:** COVID-19, Doenças Cardiovasculares, Mortalidade, Mortalidade Hospitalar

A Organização Mundial da Saúde (OMS) estima quase 15 milhões de mortes em excesso associadas à COVID-19 no mundo em 2020 e 2021, definidas como a diferença entre o número total de mortes (por todas as causas) e o número de mortes esperadas se não houvesse pandemia.^1^

No Brasil, a OMS estima 99 e 220 mortes em excesso associadas à pandemia de COVID-19 por 100.000 habitantes em 2020 e 2021, respectivamente.^1^ Isso se traduziria em cerca de 680.000 mortes a mais nos dois primeiros anos da pandemia, ou seja, dezenas de milhares a mais do que as mortes por COVID-19 oficialmente relatadas no período. Muitas dessas mortes em excesso estão relacionadas à subnotificação devido à falta de testes ou a diagnósticos incorretos (mortes verdadeiras por COVID-19 atribuídas a outras condições). Outros eventos fatais foram por outras causas e de alguma forma indiretamente associados à pandemia, como as mortes por doenças não tratadas adequadamente devido ao sistema de saúde sobrecarregado. Considerando que as doenças cardiovasculares (DCV) são a principal causa de morte no Brasil,^2^ é crucial desvendar o impacto da COVID-19 nas estatísticas de DCV.

Nesse contexto, Armstrong et al.,^3^ analisando dados de hospitais públicos no Brasil, relatam que o número de óbitos hospitalares por DCV em 2020 foi apenas 1,58% inferior ao esperado com base na média dos anos anteriores. No entanto, a taxa de letalidade hospitalar por DCV aumentou 13,3% em todo o ano e 18,8% de março a dezembro.^3^

Esses achados estão de acordo com outros estudos que relataram, durante a pandemia, redução no número de pacientes que procuram atendimento médico, diminuição das internações e procedimentos por DCV, pacientes hospitalizados mais graves e, consequentemente, aumento da letalidade hospitalar por DCV.^4–10^ É importante ressaltar que um achado repetidamente relatado é um aumento desconfortável nas mortes domiciliares.^11–13^ Portanto, agora está claro que a pandemia impactou substancialmente o cuidado com DCV no Brasil.

Quais são os aprendizados deste diagnóstico? Em primeiro lugar, espera-se que os médicos tenham aprendido que há casos em que a investigação, intervenção ou hospitalização não podem ser adiadas. Em segundo lugar, há um grande espaço para educar os pacientes sobre os sinais de alerta de condições graves, como síndrome coronariana aguda e acidente vascular cerebral, minimizando as mortes em casa devidas ao medo do paciente de ir ao hospital. Terceiro, suavizar as consequências da pandemia só é possível com um sistema de saúde bem preparado que possa responder rapidamente às demandas do surto sem comprometer o atendimento de outras doenças mortais. O Brasil não está acostumado a desastres naturais ou pandemias, e muitos subestimaram os danos potenciais do vírus. Agora nós temos a oportunidade de aprender com a experiência, como os países asiáticos fizeram com a epidemia de SARS em 2003, e melhor nos prepararmos para futuros eventos catastróficos.

Após a fase mais crítica, pré-vacinação, da pandemia, a atenção agora se volta para outra preocupação: até que ponto o cancelamento de consultas e procedimentos médicos forçados pela pandemia afetará as DCV? O controle inadequado dos fatores de risco e as intervenções realizadas tardiamente podem adicionar outra camada ao impacto da pandemia de COVID-19 nos desfechos das DCV. O monitoramento contínuo da situação é necessário e provavelmente será abordado por estudos futuros.

Outro aspecto relevante é reconhecer que os efeitos da pandemia não são uniformes na comunidade. Marinho et al.,^14^ encontraram um excesso de mortalidade de 26,3% (23,3%-29,3%) entre pretos/pardos no Brasil em 2020, enquanto esse número foi de 15,1% (14,1%-16,1%) em brancos.^14^ Em Belo Horizonte-MG, o excesso de mortalidade em 2020 aumentou à medida que o Índice de Vulnerabilidade à Saúde piorou.^15^ Além disso, Brant et al. relataram que o aumento de óbitos domiciliares por DCV em Belo Horizonte-MG em 2020 foi mais acentuado em indivíduos mais vulneráveis socialmente.^13^ Identificar os subgrupos mais afetados é estratégico para definir alvos prioritários para intervenções de saúde pública e evitar o perigoso caminho de aumento das desigualdades em saúde.

Nas últimas décadas, observamos um declínio contínuo da mortalidade por DCV ajustada por idade no Brasil, embora essa queda tenha se atenuado nos últimos anos.^2^ Ainda não está claro se a pandemia modificará substancialmente essa tendência. No entanto, a mudança no padrão de internações por DCV e o aumento inaceitável de óbitos domiciliares não podem ser assistidos passivamente sem perplexidade. É hora de aprender com os dados e agir para minimizar os impactos da pandemia nos desfechos das DCV.
