# Supplementary material for: A Pandemia de COVID-19 e a Doença Cardiovascular no Brasil: Aprendendo com os Dados
Source: Arq Bras Cardiol. 2022 Jul 7;119(1):46–7. [Article in Portuguese] doi: 10.36660/abc.20220371 (PMC9352140; doi:10.36660/abc.20220371)
Supplement: Translation

# Translation: The COVID-19 Pandemic and Cardiovascular Disease in Brazil: Learning from the Data

**Authors:** Fernando Cesena

**Affiliations:** 1 Guarulhos SP Brazil Cenocor, Guarulhos, SP – Brazil

**Keywords:** COVID-19, Cardiovascular Diseases, Mortality, Hospital Mortality

The World Health Organization (WHO) estimates nearly 15 million excess deaths associated with COVID-19 in the world in 2020 and 2021, defined as the difference between the total number of deaths (from all causes) and the number of expected deaths if there was no pandemic.^1^

In Brazil, the WHO estimates 99 and 220 excess deaths associated with the COVID-19 pandemic per 100,000 inhabitants in 2020 and 2021, respectively.^1^ This would translate into around 680,000 excess deaths in the first two years of the pandemic, *i.e.*, tens of thousands higher than the officially reported COVID-19 deaths in the period. Many of these excess deaths are related to sub notification due to lack of testing or misdiagnosis (true deaths from COVID-19 assigned to other conditions). Other fatal events were from other causes and somehow indirectly associated with the pandemic, such as the deaths from illnesses not properly treated due to the overwhelmed health system. Considering that cardiovascular disease (CVD) is the main cause of death in Brazil,^2^ it is crucial to unravel the impact of COVID-19 on CVD statistics.

In this context, Armstrong et al.,^3^ analyzing data from public hospitals in Brazil, report that the number of in-hospital deaths due to CVD in 2020 was only 1.58% lower than expected based on the average of previous years. However, the in-hospital case fatality rate due to CVD increased by 13.3% in the whole year and by 18.8% from March to December.^3^

These findings are in agreement with other studies that reported, during the pandemic, a reduction in the number of patients seeking medical care, a decrease in CVD hospitalizations and procedures, more severely diseased hospitalized patients, and consequently an increase in hospital lethality due to CVD.^4–10^ Importantly, a repeatedly reported finding is an uncomfortable increase in home deaths.^11–13^ Therefore, it is now clear that the pandemic has substantially impacted CVD care in Brazil.

What are the takeaways from this diagnosis? First, physicians are expected to have learned that there are cases where the investigation, intervention, or hospitalization cannot be postponed. Second, there is a large room to educate patients about warning signs of severe conditions, such as acute coronary syndrome and stroke, minimizing the home deaths due to patient fear of going to the hospital. Third, smoothing the consequences of the pandemic is only possible with a well-prepared health system that can rapidly respond to the outbreak's demands while not compromising the care of other deadly diseases. Brazil is not used to natural disasters or pandemics, and many underestimated the potential damage of the virus. Now we have the opportunity to learn from the experience as Asian countries did from the SARS epidemic in 2003 and better prepare ourselves for future catastrophic events.

After the most critical, pre-vaccination phase of the pandemic, the attention now shifts to another concern: to which extent will the cancellation of medical consultations and procedures forced by the pandemic affect CVD? Suboptimal risk factor control and interventions carried out late may add another layer to the impact of the COVID-19 pandemic on CVD outcomes. Continuous monitoring of the situation is needed and will probably be addressed by future studies.

Another relevant aspect is to acknowledge that the effects of the pandemic are not uniform in the community. Marinho et al.^14^ found an excess mortality rate of 26.3% (23.3%-29.3%) among blacks/browns in Brazil in 2020, while this number was 15.1% (14.1%-16.1%) in whites.^14^ In Belo Horizonte-MG, the excess mortality in 2020 increased as the Health Vulnerability Index worsened.^15^ Also, Brant et al. reported that the increase in CVD home deaths in Belo Horizonte-MG in 2020 was more pronounced in more socially vulnerable individuals.^13^ Identifying the most affected subgroups is strategic for defining priority targets for public health interventions and avoiding the dangerous path of increasing health inequalities.

In the last decades, we have observed a continuous decline in the age-adjusted CVD mortality in Brazil, although this decrease has attenuated in the last years.^2^ It is not yet clear whether the pandemic will substantially modify this trend. Nevertheless, the change in the pattern of hospitalizations for CVD and the unacceptable increase in home deaths cannot be passively watched without perplexity. It is time to learn from the data and act to minimize the impacts of the pandemic on CVD outcomes.
